# Need for cognitive closure, tolerance for ambiguity, and perfectionism in medical school applicants

**DOI:** 10.1186/s12909-020-02043-2

**Published:** 2020-04-28

**Authors:** Julia Gärtner, Lisa Bußenius, Sarah Prediger, Daniela Vogel, Sigrid Harendza

**Affiliations:** 1 III. Department of Internal Medicine, University Medical Center Hamburg-Eppendorf, Hamburg, Germany; 2 Department of Biochemistry and Molecular Cell Biology, Center for Experimental Medicine, University Medical Center Hamburg-Eppendorf, Hamburg, Germany

**Keywords:** Medical school admission, Multiple mini-interviews, Need for cognitive closure, Perfectionism, Selection for medical school, Tolerance for ambiguity, Undergraduate medical education

## Abstract

**Background:**

Physicians have to deal with uncertainty on a daily basis, which requires high tolerance for ambiguity. When medical decisions have to be made in ambiguous situations, low levels of need for cognitive closure and high levels of adaptive perfectionism are beneficial. It might be useful to measure such personality traits during medical school selection processes. In our study, we explored the expression of need for cognitive closure, tolerance for ambiguity, and perfectionism in medical school applicants who participated in a multiple mini-interview selection process with respect to the final decision of admission or rejection.

**Methods:**

After participating in the multiple mini-interview procedure (HAM-Int) at Hamburg Medical School in August 2019, 189 medical school applicants filled out a questionnaire including the Multidimensional Perfectionism Scale by Hewitt and Flett (MPS-H), the Multidimensional Perfectionism Scale by Frost (MPS-F), the Tolerance for Ambiguity Scale (TAS), the 16-Need for Cognitive Closure Scale (16-NCCS), and sociodemographic data. After the final admission decision, the scores of need for cognitive closure, tolerance for ambiguity, and perfectionism of admitted and rejected applicants were compared. We also assessed the predictive power of need for cognitive closure and age for the admission decision in a binary logistic regression model.

**Results:**

Compared to the admitted applicants, the rejected applicants showed a significantly higher need for cognitive closure (*p* = .009). A high need for cognitive closure correlated significantly positively with maladaptive perfectionism (*p* < .001) and significantly negatively with tolerance for ambiguity (*p* < .001). Low need for cognitive closure and older age were associated with a positive admission decision.

**Conclusions:**

Regarding the personality traits need for cognitive closure, tolerance for ambiguity, and perfectionism we identified interesting differences and correlations of relevance for physicians’ daily work in medical school applicants who were admitted or rejected after participating in a multiple mini-interview selection procedure. Further studies are needed to investigate these characteristics and their development longitudinally in medical students and to correlate them with students’ medical performance.

## Background

It is important for society and for future patients that medical schools accept the challenge to select the ideal people to become tomorrow’s doctors [1]. Medical students’ performance is influenced and can be predicted by personal attributes, which also predict well-being and coping strategies of medical students during the demanding medical training [2]. Knowledge-based medical school entry tests, which are frequently used, seem to select diligent applicants but might miss out on other important personal characteristics important for medical doctors [3]. A medical school admission process that additionally considers desirable personality traits which relate to performance as physicians could be advantageous in the long run [4].

Medical school selection processes which take applicants’ psychological properties into account have already been established at many medical faculties worldwide [5–7]. At the Medical Faculty of Hamburg University, the selection process for medical school applicants includes a natural science test (HAM-Nat) in a first step [8] and multiple mini-interviews (HAM-Int) in a second step [9, 10] besides high school grade point averages. We already detected that rejected applicants who participated in the multiple mini-interviews (HAM-Int) showed higher levels of depressive symptoms than admitted applicants [11]. This can be partly explained by maladaptive perfectionism [11], which is an important finding because – in contrast – adaptive perfectionism [12] has been described to be highest in medical school applicants admitted by their grade point average only [13]. Furthermore, adaptive perfectionism shows a general decrease during undergraduate medical studies independent of the admission procedure [14]. Even though adaptive perfectionism, which includes self-oriented perfectionism and personal standards [12], is an important characteristic for medical students and physicians, other personality traits are needed as well, because physicians have to endure and deal with uncertainty on a daily basis when working with patients [15, 16].

The ability to tolerate ambiguity is beneficial for being able to deal with uncertainty [17]. Individuals with lower need for cognitive closure showed increased cognitive effort for higher task ambiguity, while individuals with higher need for cognitive closure showed increased cognitive effort for higher outcome relevance [18]. This suggests that the interaction between need for cognitive closure and context influences perceptual decision making [18]. While tolerance for ambiguity describes an individual’s perception of uncertain situations, the need for cognitive closure identifies an individual’s extent of desire for closure toward ambiguity [19]. Ambiguity can be understood as an event with more than one possible interpretation and frequently occurs in medical contexts such as during history taking, diagnostics, and patient treatment. Each situation carries a varying degree of uncertainty and tolerance for ambiguity focuses on the cognitive interpretation of uncertainty [19]. An individual’s perception of an ambiguous situation can either tend to interpret it as desirable, which describes tolerance for ambiguity, or as a source of threat or discomfort, which describes intolerance of ambiguity [20, 21]. The ability to tolerate ambiguity has been shown to have an impact on physicians’ level of perceiving work-related stress [17]. Low tolerance for ambiguity is associated with the tendency to order more diagnostic tests [17] and the need for cognitive closure hampers medical decision making as well [22, 23]. Furthermore, need for cognitive closure identifies the motivation to approach or avoid closure [19] and describes an individual’s desire for a firm answer to a question and an aversion toward ambiguity [23, 24].

The relevant personal qualities for medical school students who will perform well as future physicians are not completely defined [25]. Hence, studies with medical school applicants are needed to investigate aspects which are relevant for physicians to perform well in their job for a functional health care system and to stay healthy at the same time [2, 26, 27]. Especially personality aspects like perfectionism, ambiguity tolerance, and need for cognitive closure [2, 11, 17, 28] could be desirable targets in medical student selection to measure applicants’ capacity for adapting and coping with uncertainty and decision making. Currently, these personality aspects are not included in our multiple mini-interviews, which measure psychosocial competencies such as empathy, communication skills, and self-regulation [8, 10]. We hypothesize that the expression of these personality aspects could play an additional role as prediction markers in the medical school selection process. Therefore, the aim of our study was to explore possible differences in ambiguity tolerance and in need for cognitive closure and their correlation with aspects of perfectionism between admitted and rejected medical school applicants.

## Methods

### Study design and participants

The Medical Faculty of Hamburg employs a two-step-process for the selection of medical students. First, medical applicants with a high school grade point average of 1.8 or better (a GPA of 1.0 resembles the best grade, 6.0 the worst grade) are invited to partake in a natural science test (HAM-Nat). The 100 best participants (rank 1 to 100) after the HAM-Nat are admitted to medical school, while the following 200 applicants (rank 101 to 300) are invited to participate in a mulitple mini-intervies selection process (HAM-Int). In August 2019, 196 applicants took part in the HAM-Int and were randomly divided into two groups for procedural reasons. The Hamburg Medical Faculty’s final ranking for medical school admission is based on a sum of the applicants’ GPA, HAM-Nat, and HAM-Int scores. Enrolment of study participants ensued on a voluntary basis during the debriefing phase after the HAM-Int. The questionnaire was administered to the participants who gave their written informed consent and took about 15 min to be completed. A person not involved in this study consolidated relevant admission data (e.g. HAM-Nat, HAM-Int, and GPA scores) as well as basic sociodemographic details such as gender and age with the questionnaire data. The questionnaire contained the following validated instruments: Multidimensional Perfectionism Scale by Hewitt and Flett (MPS-H) [29], Multidimensional Perfectionism Scale by Frost (MPS-F) [30], Tolerance for Ambiguity Scale [20], and the 16-Need for Cognitive Closure Scale [24]. Applicants signed informed consent prior to the admission procedure and the local psychological ethics commission (Lokale Psychologische Ethikkommission am Zentrum für Psychosoziale Medizin – LPEK) approved of this study (reference number: LPEK-0042) under the condition that data were anonymized. We clearly stated that any results of the questionnaire would not affect the HAM-Int score. The final admission decision was still unknown to the participants and the authors of this study at the time when the questionnaire was filled out.

### Instruments

#### Multidimensional perfectionism scale (MPS-H)

The MPS-H consists of three scales, each containing 15 items: Self-oriented perfectionism (SOP), Other-oriented perfectionism (OOP), and Socially-prescribed perfectionism (SPP). Items are rated on a 7-point Likert scale (1 = full disagreement to 7 = full agreement) [29]. We used the German version of this instrument [11, 13] based on Stoeber [31]. The three scales showed satisfactory internal consistency (Cronbach’s alpha = .88, .84 and .74 respectively).

#### Multidimensional perfectionism scale (MPS-F)

This instrument consists of a total of 35 items rated on a 6-point Likert scale (1 = being not at all true to 6 = being exactly true) [30] (German version by Altstötter-Gleich and Bergemann [32]). This scale measures six dimensions, i.e. Personal standards (PS, seven items), Organization (O, six items), Concern over mistakes (CM, nine items), Doubts about actions (DA, four items), Parental expectations (PE, five items), and Parental criticism (PC, four items). Five scales showed acceptable to good internal consistency (Cronbach’s alpha for O = .92, PE = .87, CM = .84, PS = .75, and PC = .74), while the reliability of the scale DA only showed an internal consistency of .54.

#### Tolerance for ambiguity scale (TAS)

This scale originates on the measure of tolerance for ambiguity by Herman et al. [20] as an improved variant of the Budner scale [21]. The TAS refined the Budner scale to 12 items covering the four dimensions Valuing diverse others, Change, Challenging perspectives, and Unfamiliarity [20]. The items are scored on a 5-point Likert scale (1 = strongly disagree, 3 = neither agree nor disagree, 5 = strongly agree). The items were translated into German by JG and the final versions were discussed with SH, SP, and DV. It shows an internal consistency of .68.

#### 16-need for cognitive closure scale (16-NCCS)

The 16-NCCS [24] comprises 16 items and measures the need for cognitive closure. The 16-item NCC scale is an advancement of the 15-item NFC scale [33], which was constructed from an earlier 47-item version to measure the need for closure [23]. The 16-NCCS is valid for the use in German language [24]. The instruments’ internal consistency is .79.

### Data processing

Optical mark recognition software (Remark Office OMR, pki Informationssysteme für Marktforschung, Hamburg, Germany) was used to obtain data from the questionnaires. Data were analyzed with a general alpha-level of .05 using IBM SPSS Statistics for Windows, version 25 (IBM Corp., Armonk, N.Y., USA). GPA and all scores from the selection process were transformed into a linear scale so that the GPA accounts for 34% of the final score, while HAM-Nat and HAM-Int account for 33%, respectively. HAM-Nat and HAM-Int scores ranged from 0 to 59, while GPA ranged from 0 to 60. We included an individual participant in the data set when at least 80% of the questionnaire items were rated. Imputation of the mean of the scale ensued for remaining missing data. Sum scores from the subscales of the MPS-H as well as from the MPS-F were calculated and *z*-standardized. The composite measure of adaptive perfectionism (AP) consists of the *z*-standardized scores of SOP from MPH-S and PS from MPH-F, while Maladaptive Perfectionism (MP) consists of the *z*-standardized subscales SPP from MPH-S, and CM and DA from MPH-F. Group differences between admitted and rejected applicants were calculated with independent *t*-tests. Where applicable, Cohen’s *d* was calculated to estimate effect sizes. The general association of adaptive as well as maladaptive perfectionism, tolerance for ambiguity, and need for cognitive closure as well as their association with the decision measures was assessed using Pearson correlations. We calculated a binary logistic regression model to predict the admission decision with the predictors of need for cognitive closure, age, tolerance for ambiguity, adaptive perfectionism, and maladaptive perfectionism.

## Results

Of 196 administered questionnaires, 189 questionnaires were returned. Five of these had to be excluded before data processing due to missing identification and 11 had to be excluded because of missing written consent for data consolidation. A total of 173 questionnaires entered data processing (return rate: 88%). Participants’ characteristics are displayed in Table 1. Mean age of medical school applicants was 19.90 ± 1.63 years, with a minimum age of 17 and a maximum age of 25 years. Mean GPA was 1.51 ± 0.19 (mean GPA score: 49.71 ± 3.77), mean HAM-Nat score was 35.55 ± 4.20, and mean HAM-Int score was 34.82 ± 4.97. Mean total score, on which the applicants’ final ranking was based, was 120.09 ± 5.58. Applicants showed a mean 16-NCCS score of 2.89 ± 0.59, a mean TAS score of 3.55 ± 0.48, a score of .01 ± 1.79 for adaptive perfectionism (AP) and a score of −.02 ± 2.45 for maladaptive perfectionism (MP). Of the 173 applicants, 88 were eventually admitted according to the acceptance quota, while 85 were rejected. Rejected applicants were significantly younger (*p* = .005, *d* = 0.43), had significantly lower HAM-Nat scores (*p* = .013), significantly lower HAM-Int scores (*p* < .001, *d* = 1.94), and significantly lower total scores (*p* < .001, *d* = 2.47). Rejected applicants showed a significantly higher score on the 16-NCCS (*p* = .009). No significant differences between rejected and admitted applicants in GPA, GPA score, MPS-H or MPS-F as well as TAS scores were found.

**Table 1:** Participants’ characteristics

**Sociodemographic data**	Applicants (total)	Admitted applicants	Rejected applicants	Cohen’s ***d***
N	173	88	85	
Sex (female/male)	120/53	64/24	56/29	
Age (mean ± SD)	19.90 ± 1.63	20.24 ± 1.60	19.55 ± 1.60**	0.43
**Selection criteria** (mean ± SD)				
GPA	1.51 ± 0.19	1.51 ± 0.18	1.52 ± 0.20	0.05
GPA score	49.71 ± 3.77	49.80 ± 3.56	49.62 ± 3.98	0.05
HAM-Nat score	35.55 ± 4.20	36.32 ± 3.77	34.75 ± 4.49**	0.38
HAM-Int score	34.82 ± 4.97	38.23 ± 2.95	31.31 ± 4.11***	1.94
Total score	120.09 ± 5.58	124.35 ± 3.17	115.68 ± 3.82***	2.47
**Personality measures** (mean ± SD)				
16-NCCS	2.89 ± 0.59	2.77 ± 0.59	3.01 ± 0.57**	0.40
TAS	3.55 ± 0.48	3.57 ± 0.47	3.53 ± 0.49	0.09
Adaptive Perfectionism (AP)	.01 ± 1.79	0.14 ± 1.81	-0.13 ± 1.77	0.15
Maladaptive Perfectionism (MP)	-.02 ± 2.45	-0.23 ± 2.37	0.22 ± 2.54	0.18

*GPA* high school grade point average (best score: 1.0; worst score: 6.0), HAM-Nat = Hamburg Assessment Test for Medicine – Natural Science Test, HAM-Int = Hamburg Assessment Test for Medicine – Interview, *16-NCCS* 16-item Need for Cognitive Closure Scale, *TAS* Tolerance for Ambiguity Scale, **: *p* < .01, ***: *p* < .001

The 16-NCCS correlated highly negatively with the TAS (*r* = −.57, *p* < .001), highly positively with MP (*r* = .51, *p* < .001), and moderately positively with AP (*r* = .29, *p* < .001). The TAS showed a moderate negative association with MP (*r* = −.29, *p* < .001), but not with AP. The correlation between admission measures and questionnaires’ variables only showed a significant weak positive correlation between HAM-Int and 16-NCCS (*r* = .182, *p* = .017) (Table 2). The logistic regression model with the variables need for cognitive closure, tolerance for ambiguity, adaptive perfectionism, maladaptive perfectionism and age (Table 3) was statistically significant (χ^2^(5) = 17.81, *p* = .003). The model correctly classified 57% of cases and explained 13.5% (Nagelkerke’s *R*^2^) of the variance in admission decisions. Older age and lower need for cognitive closure were associated with an increased likelihood of a positive admission decision. Tolerance for ambiguity, adaptive perfectionism or maladaptive perfectionism did not significantly predict the admission decision in the logistic regression model.

**Table 2:** Correlation of perfectionism, need for cognitive closure, tolerance for ambiguity, GPA, HAM-Nat, and HAM-Int

	Adaptive Perfectionism	Maladaptive Perfectionism	16-NCCS	TAS	GPA	HAM-Nat	HAM-Int
**Adaptive Perfectionism**	1	.530***	.290***	-.106	-.102	-.092	.085
**Maladaptive Perfectionism**		1	.508***	-.287***	.008	.030	-.105
**16-NCCS**			1	-.571***	.103	.049	-.182*
**TAS**				1	-.040	-.058	.101
**GPA**					1	.810***	.052
**HAM-Nat**						1	.052
**HAM-Int**							1

*16-NCCS* 16-item Need for Cognitive Closure Scale, *TAS* Tolerance for Ambiguity Scale, *GPA* high school grade point average, *HAM-Nat* Hamburg Assessment Test for Medicine – Natural Science Test, *HAM-Int* Hamburg Assessment Test for Medicine – Interview, *: *p* < .05, ***: *p* < .001

**Table 3:** Binary logistic regression analysis for prediction of admission decision

Parameter	Regression coefficient	Standard error	***Wald***	df	***p***	Odds ratio
16-NCCS	-1.09	0.40	7.27	1	.007	.34
Age	0.28	0.10	7.21	1	.007	1.33
AP	0.33	0.20	2.75	1	.097	1.39
TAS	-0.61	0.44	1.91	1	.167	.55
MP	-0.09	0.22	0.17	1	.678	.91
**Model fit**	**Nagelkerke’s*R***^**2**^		**χ**^**2**^	**df**	***p***	
	.135		17.81	5	.003	

*16-NCCS* 16-item Need for Cognitive Closure Scale, *AP* Adaptive Perfectionism, *TAS* Tolerance for Ambiguity Scale, *MP* Maladaptive Perfectionism

## Discussion

Multiple mini-interviews are designed to select applicants with personality traits which are supposed to predict that they will become “good doctors” [7]. Rejected applicants who participated in our multiple mini-interview medical school selection procedure had a higher need for cognitive closure compared to admitted applicants. A higher need for cognitive closure can lead to lower decision quality in medical practice [34] and seems to be a less desirable personality trait for physicians. Therefore, the need for cognitive closure could be measured during the selection process as additional prediction marker for desired applicants. Individuals with lower need for cognitive closure invest more cognitive effort when tasks become more difficult [18], seem to be more desirable to be selected for medical studies because physicians have to deal with increasing difficulty of tasks on a daily basis.

Furthermore, in our study, a higher need for cognitive closure, which we predominantly found in rejected applicants, was related to lower tolerance for ambiguity. However, in physicians’ diagnostic processes, high tolerance for ambiguity is needed – and therefore desirable in medical school applicants, because the majority of patients present with complaints that cannot be instantly classified as distinct disease entities [35]. Furthermore, low tolerance of ambiguity is related to lower tolerance of uncertainty [36], which also frequently occurs in medical practice and needs to be handled professionally. Hence, an aversion towards ambiguity [37] is not supportive for future physicians’ daily work and does not seem desirable in medical school applicants. Hence, measuring the tolerance of ambiguity in medical school applicants could be an interesting additional aspect in the selection process to predict suitable applicants for undergraduate medical studies.

Additionally, higher levels of intrinsic motivation have been linked to lower levels of need for cognitive closure and vice versa [18]. In our study, maladaptive perfectionism, which is composed of the subscales Socially-prescribed perfectionism, Concern over mistakes, and Doubts about action [12], was also related to higher need for cognitive closure which confirms these findings and does not seem to be a desirable personality trait for medical students or physicians. Furthermore, we could show in a previous study that undergraduate medical students with higher levels of maladaptive perfectionism showed higher scores for symptoms of depression and anxiety [13]. These findings suggest, that measuring the different aspects of perfectionism in addition to the need for cognitive closure and the tolerance of ambiguity could support finding the desired medical school applicants in the selection process.

Our binary logistic regression model showed lower need for cognitive closure and older applicants’ age as main predictors for the admission decision. The admission measures seem to assess the constructs we investigated in this study, but the bivariate correlation only revealed the 16-NCCS as weakly correlating with the multiple mini-interviews. The students’ age seemed to have the main impact on admission measures. However, students’ age should not be a discriminatory factor for admission decisions. Interestingly, older age was associated with higher need for cognitive closure in another study but the efficacy of fulfilling the need for cognitive closure decreased with aging [38]. Hence, the higher need for cognitive closure in older individuals does not necessarily result in the same closed behaviour found by younger individuals with need for cognitive closure [39]. Furthermore, the need for cognitive closure level of an individual is only consistent with the individual’s behaviour under positive mood [40]. These findings show that measuring an individual’s need for cognitive closure alone might not be sufficient to predict a person’s actual behaviour. A higher level of tolerance for ambiguity and perfectionism was found in individuals above 25 years of age [2]. Interestingly, ambiguity aversion, which positively correlates with the need for cognitive closure [24], also increased with older age [37]. Being able to have high tolerance for ambiguity helped medical students to cope with uncertainty [41] and was also shown to play a role in medical school selection [42].

A strength of our study is the differentiated view on medical school applicants for dealing with uncertainty by studying the combined expressions of need for cognitive closure, tolerance for ambiguity, and perfectionism in a large cohort of medical school admitted and rejected applicants who participated in a multiple mini-interview admission procedure. Both perfectionism scales showed good internal consistency and the results were similar to our previous study with another cohort of medical school applicants [11]. The 16-NCCS showed an acceptable internal consistency of .79. A limitation of our study is the TAS’ low reliability of .68. This could be due to item translation without additional validation of the translated questionnaire. Another limitation is that this study was only performed at one institution and that only a single admission process was observed with one cohort of applicants. However, our findings still provide a first assumption of association between these characteristics in medical school applicants, which need to be further investigated.

Our results underscore that measuring the need for cognitive closure alone and possibly rejecting medical school applicants with a higher need for cognitive closure, because it can lead to lower decision quality in medical practice [34], could comprise the risk of neglecting useful abilities in individuals with higher need for cognitive closure [18], which could be needed in certain medical specialties. Measuring the different aspects of perfectionism in combination with the need for cognitive closure in medical school applicants like in our study provides a possibility to identify the status of applicants’ motivation. Since motivation is related to mood [43] and the level of need for cognitive closure and an individual’s actual behaviour are consistent under positive mood [40], an additional test for medical school applicants’ mood might help to predict their future behaviour as physicians. Similar to a study in physicians [17] it might be useful to test medical students with respect to these personality traits longitudinally to assist them in developing their personal potential. Since higher need for cognitive closure leads to a preference to consider smaller amounts of information before making final decisions [44], which might hamper decision-making processes in the long run, teaching strategies and exercises need to be explored that reduce the need for cognitive closure [45].

## Conclusions

Our study shows different personality aspects with respect to the need for cognitive closure in medical school applicants participating in a multiple mini-interview selection test. Need for cognitive closure was significantly higher in rejected applicants and higher need for cognitive closure was related to maladaptive perfectionism and to lower tolerance for ambiguity. A lower score of need for cognitive closure was a main predictor for the admission decision in combination with older age. With respect to medical decision-making of physicians in an environment of great uncertainty, the admission of applicants with a profile of low need for cognitive closure, high tolerance for ambiguity, and low levels of maladaptive perfectionism seems desirable. Since an individuals’ need for cognitive closure can be modulated by their ability to achieve cognitive closure, the latter seems to be an additional important measure. To decide whether testing medical school applicants for these different personality traits could be useful or whether different admission selection procedures already implicitly select students with respect to these measures, further research is necessary. Longitudinal studies in admitted medical students for such characteristics would additionally be interesting to monitor their possible changes and to combine the levels of their expression with medical students’ performance.

## Data Availability

All data and materials are available from the manuscript and from the corresponding author upon request.

## Abbreviations

AP: Adaptive perfectionism
CM: Concern over mistakes
DA: Doubts about actions
GPA: High school grade point average
HAM-Int: Hamburg Assessment Test for Medicine – Interview
HAM-Nat: Hamburg Assessment Test for Medicine – Natural Science Test
MP: Maladaptive perfectionism
MPS-F: Multidimensional Perfectionism Scale by Frost
MPS-H: Multidimensional Perfectionism Scale by Hewitt and Flett
NFC: Need for cognitive closure
16-NCCS: Need for Cognitive Closure Scale
O: Organization
OOP: Other-oriented perfectionism
PC: Parental criticism
PE: Parental expectations
PS: Personal standards
SOP: Self-oriented perfectionism
SPP: Socially-prescribed perfectionism
TAS: Tolerance for Ambiguity Scale
